# Feasibility of home-based ELISA capillary blood self-testing for anti-SARS-CoV-2 antibodies

**DOI:** 10.1016/j.plabm.2022.e00290

**Published:** 2022-07-12

**Authors:** Stéphanie Baggio, Giuseppe Togni, Isabella Eckerle, Nicolas Vuillemier, Laurent Kaiser, Laurent Gétaz

**Affiliations:** a Division of Prison Health, Geneva University Hospitals and University of Geneva, Geneva, Switzerland; b Institute of Primary Health Care (BIHAM), University of Bern, Bern, Switzerland; c Microbiology Lab, Unilabs, Coppet, Switzerland; d Department of Molecular Medicine and Microbiology, Faculty of Medicine, University of Geneva, Geneva, Switzerland; e Center for Emerging Viral Diseases, Geneva University Hospitals and Faculty of Medicine, Geneva, Switzerland; f Division of Infectious Diseases, Department of Medicine, Geneva University Hospitals and Faculty of Medicine, Geneva, Switzerland; g Laboratory of Virology, Division of Laboratory Medicine, Geneva University Hospitals and Faculty of Medicine, Geneva, Switzerland; h Division of Laboratory Medicine, Department of Diagnostics, Geneva University Hospitals and Faculty of Medicine, Geneva, Switzerland; i Division of Laboratory Medicine, Department of Medical Specialties, Geneva University Hospitals and Faculty of Medicine, Geneva, Switzerland; j Department of Medicine, Faculty of Medicine, University of Geneva, Switzerland; k Division of Tropical and Humanitarian Medicine, Geneva University Hospitals, Geneva, Switzerland

**Keywords:** Antibodies, COVID-19, SARS-CoV-2, Serological testing, SARS-CoV-2, Severe acute respiratory syndrome coronavirus 2, Covid-19, Coronavirus disease of 2019, ELISA, Enzyme-linked immunosorbent assays, RT-PCR, Real-time reverse transcription–polymerase chain reaction, IgG, Immunoglobulin G, OR, Odd-ratio

## Abstract

**Objectives:**

Serological assays for the presence of anti-SARS-CoV-2 antibodies are crucially needed for research and monitoring of the SARS-CoV-2 pandemic. Antibodies are reliability detected in capillary blood, a minimally invasive and cost-effective alternative to venous blood testing. However, there is a limited knowledge on feasibility of capillary blood self-sampling. This study compared the feasibility of capillary blood self-testing in people aged less than 65 *vs.* people aged 65 or more. A secondary aim was to investigate the performance of the Hem-Col® (no additive) device compared to venous blood testing.

**Design and methods:**

Data were collected in a prospective study in Switzerland (n = 106). Capillary blood was collected using the Hem-Col® (no additive) device. Feasibility was assessed using 1) collecting the recommended amount of capillary blood and 2) achieving all steps of capillary blood collection. A sample of 5 ml of venous blood was also collected.

**Results:**

For the primary objective, 86.2%/62.1% of patients aged less than 65 collected the recommended amount of capillary blood/achieved all steps *vs.* 62.5%/39.6% of patients aged 65 or more (p = .006/p = .022). For the secondary objective, the correlation between capillary and venous blood was r = 0.992 and kappa = 1.

**Conclusions:**

Capillary blood self-testing appeared as a feasible and reliable alternative to venous blood testing. Such alternative would improve access to serological testing and spare health care resources. However, the difference between age groups should be considered when using self-sampling devices. Help should be developed for older people, such as phone counseling or encouraging asking younger family members for help.

## Highlights

• Evidence of the feasibility of capillary blood self-testing is scarce.
• Capillary blood self-testing appeared as reliable and feasible alternative.
• Older people (aged 65 or more) were more likely to fail than younger people.

## 1 Introduction

The SARS-CoV-2 pandemic has now hit almost all countries over the world and vaccines are available. Public health responses and scientific knowledge on SARS-CoV-2 and Covid-19 have improved, however research and monitoring are still crucially needed. Serological essays for the presence of anti-SARS-CoV-2 antibodies are therefore critical. Indeed, they are needed for clinical studies to understand antibody responses and immunity after illness and vaccine-induced responses [1]. They are also useful for surveillance purposes to monitor the pandemic and to guide public health decisions, especially because an important proportion of SARS-CoV-2 cases are asymptomatic and therefore not detected [2,3].

Detection of anti-SARS-CoV-2 antibodies has long required collection of venous blood. Recent studies reported that antibodies are reliably detected in capillary blood [1,2]. Capillary blood testing is minimally invasive and could be a cost-effective alternative to venous blood testing. It has several benefits to improve access to serological testing and to reduce risks, including facilitating access to hard-to-reach populations, limiting the burden of serological testing, decreasing risk of infection due to in-person settings, and sparing health care resources [1,2,4]. To date, it has been successfully used in large-scale surveys [[5], [6]], among children [7], elderly [8], and vulnerable populations [9,10]. Self-testing is therefore useful for patients who would forego care for fear of transmission in medical facilities including blood collection centers.

Previous studies have tested the performance of capillary blood testing compared with venous blood, but few studies investigated feasibility of anti-SARS-CoV-2 antibodies self-testing [2]. Therefore, there is a limited knowledge on the concrete usefulness of this kind of self-testing and more research on this topic is needed [1]. To our knowledge, only three studies focused on this important topic. Two of them used unsupervised settings [11,12], meaning that feasibility was informed using self-reports and was not reliably assessed by external trained supervisors. Both studies reported good feasibility (∼91%). Another study used a supervised setting [13], with participants performing a dried blood spot test in front of a trained healthcare professional. They concluded that the test was adapted to the general population, with 100% of the participants managing to correctly do the test with only 14.5% asking for verbal help. These three feasibility studies all used rapid lateral flow immunoassays (LFIA) [[11], [12], [13]]. However, enzyme-linked immunosorbent assays (ELISA) have been demonstrated superior to other technologies and capillary blood can be reliably used to assess anti-SARS-CoV-2 antibodies status [1]. Capillary blood self-sampling and postage to laboratories appears as a good alternative to in-person venous blood testing. Information on the feasibility of ELISA self-tests is therefore still needed.

This study thus investigated the feasibility of a capillary blood self-sampling in the community. As older people are especially concerned by in-home testing to avoid risks of infection and because they can have a limited access to testing facilities, we compared the feasibility of self-testing for people aged less than 65 *vs.* people aged 65 or more. A secondary aim was to investigate the performance of the serology for anti-SARS-Cov2-antibodies collecting blood with a specific capillary self-sampling, the Hem-Col® (no additive) device (Labonovum, Limmen, the Netherlands) compared to venous blood testing.

## 2 Materials and methods

### 2.1 Study design and participants

We conducted a prospective single-center cross-sectional study in the Geneva University Hospitals, located in Geneva, Switzerland. Data were collected in May 2020, at the end of the first wave of the pandemic.

Participants were included if 1) they were aged 18 years or more, 2) had a positive RT-PCR, and 3) had Covid-19 symptoms at least four weeks before study inclusion. Exclusion criteria were 1) absence of capacity of discernment, 2) not living in the Canton of Geneva, and 3) not able to communicate in French. All participants provided written informed consent before study participation. The Cantonal Ethics Research Committee of Geneva approved the study protocol (no. 2020-00931).

### 2.2 Procedures

Eligible participants were selected from the database of SARS-CoV-2 positive patients at the outpatient SARS-CoV-2 test center [14,15]. Patients aged 65 or more were systematically invited to participate whereas patients aged less than 65 were randomly selected. Patients were invited to participate by phone until the desired sample size was reached. Those who agreed to participate were either invited to come to a medical diagnostic lab in Geneva or a nurse came for an in-home visit.

The unique visit had the following procedure: First, participants received information on the study and signed the consent form. Second, they answered a sociodemographic and clinical questionnaire, presented by a trained nurse. Third, they used the capillary blood self-test under the supervision of the nurse, after reading the instructions for use and seeing a video explaining how to use the self-test. The video complemented written instructions to help people with lower literacy skills. The nurse did not intervene to help the patient. The nurse recorded whether they managed to use the self-test. Participants then answered questions related to their satisfaction with the self-test. Finally, the nurse collected a sample of venous blood.

### 2.3 Biological material

Capillary blood was collected using Hem-Col® (no additive) device according to the recommendations of the capillary tube supplier (Labonovum, Limmen, the Netherlands). Capillary blood samples collected with this device are stable for serologic analysis for 3–5 days. Five ml of venous blood was also collected and centrifuged 10 min at 2000 g. Sera were separated from freshly collected blood and processed in the laboratory within 24 h. We assessed anti-SARS-CoV-2 IgG antibodies using an ELISA (Euroimmun; Lübeck, Germany) targeting the S1 domain of the spike protein on serum of capillary and venous blood.

### 2.4 Measures

The feasibility was assessed using two different variables: collecting the recommended amount of capillary blood and achieving all steps of capillary blood collection.

*Correct capillary blood collection*. The nurse registered whether the participants collected the recommended amount of blood (at least 240 μl corresponding to six drops of blood, corresponding to step no. 5 below). The information was recorded as a binary variable (yes/no).

*Steps of capillary blood collection*. Seven steps were recorded by the nurse: 1) hand warming, 2) finger massage, 3) remove the cap of the lancet, 4) sufficiently press the lancet against the finger, 5) collect the recommended amount of blood, 6) correctly close the tube, and 7) shake the tube. We computed a total score ranging from 0 to 7 and a binary variable assessing whether participants achieved all steps (yes/no). Three additional steps not mandatory for correct capillary blood collection were also recorded: finger disinfection, finger drying, and put a plaster.

*Other variables*. We collected gender and presence of chronic somatic diseases (cardiovascular diseases, cancer, diabetes, immunological diseases, kidney diseases, respiratory diseases, obesity, and rheumatologic diseases). Participants also answer whether they were satisfied with the collection of the blood sample (recoded as yes/no).

### 2.5 Statistical analyses

With power = 0.8, alpha = 0.05, prevalence estimates of recommended blood collection of 92% among participants aged less than 65 (unpublished data from the manufacturer) and an estimate of 70% among participants aged 65 or more, the needed sample size was n = 96. We therefore aimed to include at least n = 48 in each group.

We first computed descriptive statistics, using proportions and means/standard deviations according to the type of variables. For the primary objective, we then compared the two groups (aged less than 65 *vs.* 65 or more) using unadjusted and adjusted logistic and negative binomial regressions. The outcomes were 1) correct capillary blood collection, 2) achieving all steps of capillary blood collection, and 3) number of achieved steps of capillary blood collection. Adjusted analyses controlled for gender and presence of any chronic disease. For the secondary objective, we computed a Pearson correlation between continuous measures of the capillary blood and venous blood and the overall agreement (Kappa) between measures.

## 3 Results

**Fig. 1:**
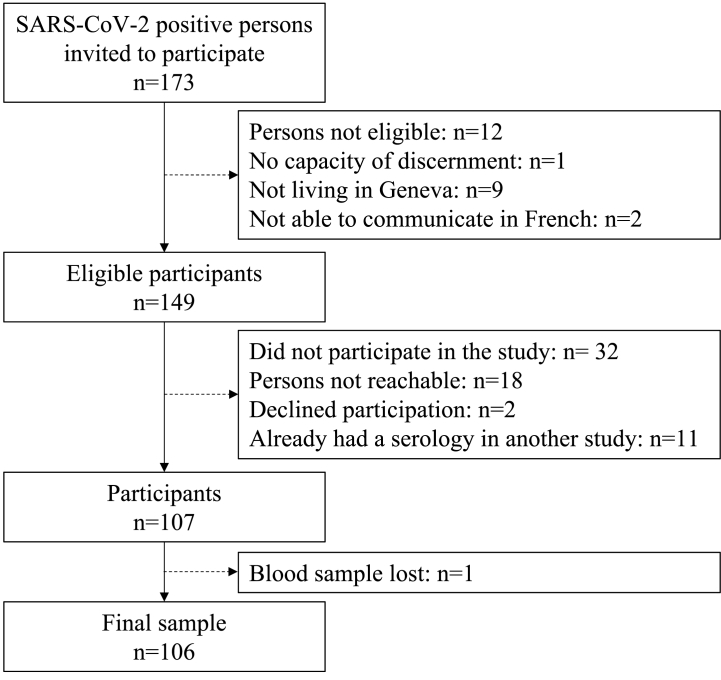
Flow chart.

A total of 106 patients (n = 48 aged 65 or more, n = 58 aged less than 65) were included in the study, with a participation rate of 78% (see Fig. 1 for the detailed flow chart). A total of n = 105 blood samples were analyzed (one sample was lost in the group of less than 65). Preliminary results are reported in Table 1. Participants in the group 65 or more were more likely to be male (p = .003), whereas there was a similar proportion of participants having at least one chronic disease in both groups (p = .428).

**Table 1:** Descriptive statistics and comparisons between groups.

Variables	Age <65	Age ≥65	Odd-ratio	p-value
	n = 58	n = 48		
Age^a^	45.7 (12.1)	70.6 (5.4)	24.82	<.001
Gender^b^				
Female	60.3 (35)	31.3 (15)	3.35	.003
Male	39.7 (23)	68.7 (33)		
Any chronic disease^b^	77.6 (45)	70.8 (34)	1.43	.428
Cardiovascular disease^b^	13.8 (8)	47.9 (23)	–	–
Cancer^b^	1.7 (1)	4.2 (2)	–	–
Diabetes^b^	6.9 (4)	20.8 (10)	–	–
Immunological disease^b^	12.1 (7)	6.3 (3)	–	–
Kidney disease^b^	8.6 (5)	4.2 (2)	–	–
Respiratory disease^b^	46.6 (27)	10.4 (5)	–	–
Obesity^b^	24.1 (14)	22.9 (11)	–	–
Rheumatologic disease^b^	10.3 (6)	14.6 (7)	–	–
Capillary blood collection				
Hand warming^b^	94.8 (55)	89.6 (43)	–	–
Finger massage^b^	87.9 (51)	70.8 (34)	–	–
Remove lancet cap^b^	98.3 (57)	93.4 (45)	–	–
Press lancet^b^	89.7 (52)	79.2 (38)	–	–
Collect recommended amount of blood^b^	86.2 (50)	62.5 (30)	0.27	.006
Close tube^b^	91.4 (53)	68.8 (33)	–	–
Shake tube^b^	82.8 (48)	66.7 (32)	–	–
No. of achieved steps^c^	6.3 (1.2)	5.3 (1.9)	−1.17	.035
Achieved all steps^b^	62.1 (36)	39.6 (19)	0.40	.022
Finger disinfection^b^	98.3 (57)	93.8 (45)	–	–
Dried finger^b^	89.7 (52)	85.4 (41)	–	–
Put a plaster^b^	89.7 (52)	70.8 (34)	–	–
Satisfaction with capillary blood collection^b^	75.4 (43)	71.7 (33)	0.83	.671

a Means and standard deviations, b estimate and p-value for unadjusted linear regressions.

b Percentages and n, odd-ratio and p-values for unadjusted logistic regressions.

c Means and standard deviations, b estimate and p-value for unadjusted negative binomial regressions.

For the primary objective, 86.2% of participants aged less than 65 and 62.5% of participants aged 65 or more were able to collect the recommended amount of capillary blood (i.e., at least 240 μl). All samples were nonetheless analyzable. The difference between groups was statistically significant (odd-ratio (OR) = 0.27, p = .006). Similarly, participants aged less than 65 were more likely to achieve all steps of capillary blood collection (62.1%) compared to participants aged 65 or more (39.6%, OR = 0.40, p = .022). Participants aged less than 65 achieved on average 6.3 steps against 5.3 for participants aged 65 or more (p = .035). Detailed statistics for each step are reported in Table 1. In analyses adjusted for age and presence of any chronic illness, age groups were significantly associated with collecting recommended amount of capillary blood (OR = 0.26, p = .007), achieving all steps of capillary blood collection (OR = 0.35, p = .014), and number of achieved steps of capillary blood collection (estimate = −0.20, p = .021).

**Fig. 2:**
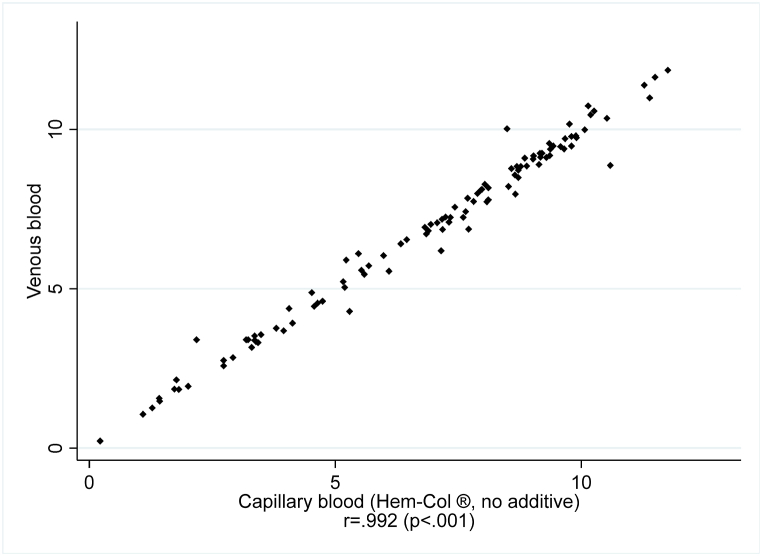
Plot of anti-SARS-CoV-2 IgG antibodies using an ELISA targeting the S1 domain of the spike protein: capillary blood (Hem-Col ® (no additive) device) against venous blood.

Regarding the secondary objective, there was a concordance of 100% between venous and capillary blood (kappa = 1, 104 samples had anti-SARS-CoV-2 antibodies out of 105 samples, 98.1%). Samples with less than 240 μl of capillary blood also yielded concordant results with venous blood. Correlations between continuous measures are reported in Fig. 2. The Pearson correlation was r = 0.992 (p < .001) and r = 0.991 (p < .001) after excluding participants who did not collect enough capillary blood.

## 4 Discussion

### 4.1 Main findings

The first objective of the study was to compare the feasibility of a capillary blood self-sampling among people aged less than 65 and people aged 65 or more. Our findings showed that a majority of people were able to perform capillary self-sampling with the Hem-Col® (no additive) device, in accordance with previous studies using other devices [[11], [12], [13]]. However, older people were 1.3 time more likely to fail compared to younger patients: 62.5% of people aged 65 or more collected enough capillary blood, in comparison with 86.2% of people aged less than 65. When considering the achievement of all steps of capillary blood collection described in the written procedure and the explanatory video, there were 62.1% of patients aged less than 65 and 36.9% of patients aged 65 or more who were successful, with older patients being 1.5 times more likely to fail compared to younger patients. Therefore, the difference between age groups should be considered when using self-sampling devices. For older people who failed to collect enough blood despite written instructions and the explanatory video, other sources of help should be developed. For example, planning a telephone counseling with a laboratory technician or encouraging the patient to ask a younger family member for help would increase the chance of collection enough capillary blood.

Even if not all participants were able to collect enough blood, all capillary blood sample were analyzable, meaning that capillary blood testing is a feasible alternative to venous blood testing. It should be considered as a valuable alternative to improve access to serological testing, useable among hard-to-reach populations and people reluctant to venous blood testing [1,2,4]. It can also decrease use of health care service, therefore sparing resources and decreasing the risk of SARS-CoV-2 infection due to in-person settings [1,2,4]. Moreover, self-collected sampling can increase the acceptance rate of screening in hidden high-risk populations [16]. We should remember that capillary blood self-testing does not replace medical consultation. For patients who do not consult their doctor and do not go to a blood collection center because of the risk of transmission, telephone or telemedicine consultations represent alternative to get advice from their physician [17]. Depending on the analyses (type and number), the volume of serum needed varies. In this study, 10 μl were enough to perform the analysis. This explains why capillary testing was feasible even if a notable proportion of patients were not able to collect 240 μl.

The secondary aim was to investigate the performance of the Hem-Col® (no additive device compared to venous blood testing. Self-sampling appeared as a very reliable tool, as there was an excellent concordance between venous and capillary blood for anti-SARS-CoV-2 antibody measures. These findings are consistent with previous reports concluding that antibodies are reliably detected in capillary blood [1,2]. Contrariwise to a rapid point-of-care LFIA, a device used to confirm the presence or absence of a target analyte, auto-collection of capillary blood in microtubes has important advantages. The analysis of several markers is possible with the Hem-Col® (no additive) device, which make its use promising for both individual testing and large-scale surveys. With some analytical devices, such as the Euroimmun analyser used in this study, 10 μl of serum (analytic volume) are enough for a quantitative determination of each marker. For SARS-Cov-2, a quantitative assay of anti-Spike 1 and anti-nucleocapsid antibodies is feasible to differentiate between immunity acquired by vaccination or infection, and to estimate the antibody level by quantitative assay.

### 4.2 Limitations

The study has, however, some limitations. Study participants might not be representative of the general adult population, as the participants were mostly people with comorbidities. However, few medical conditions are not expected to influence the results of the study and comorbidities were included as a confounder in adjusted analyses. Larger studies focusing on other groups of the population (e.g., hard-to-reach populations, individuals with a limited health literacy) are also needed to better understand how capillary blood testing could improve access to serological testing.

## 5 Conclusions

To conclude, capillary blood testing using ELISA appeared as a feasible and reliable alternative to venous blood testing and could be used for surveillance and research purposes, for anti-SARS-CoV-2 antibodies, but also potentially for serology for other diseases. Being cost-effective and minimally invasive, it would improve access to serological testing.

## Authors’ contributions

Conceptualization: SB, GT, IE, NV, LK, and LG; Formal analysis: SB; Funding acquisition: LG; Investigation: GT and LG; Methodology: SB, GT, IE, NV, LK, and LG; Project administration: SB and LG; Supervision: LG; Writing - original draft: SB; Writing - review & editing: GT, IE, NV, LK, and LG.

## Funding

This work was supported by the Fondation privée des 10.13039/501100006388HUG, Geneva, Switzerland.

## Declaration of competing interest

None.
